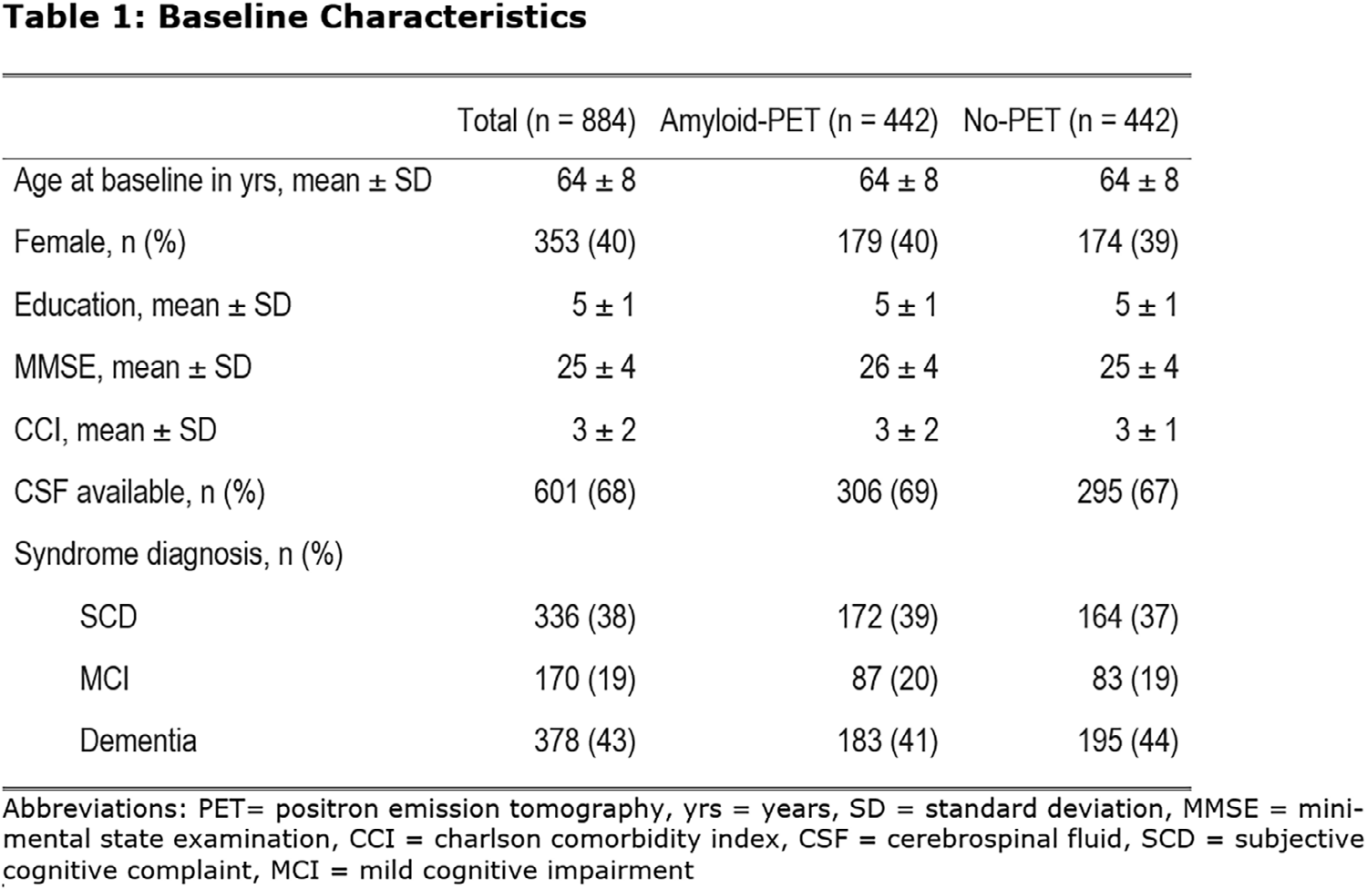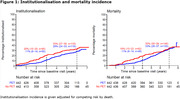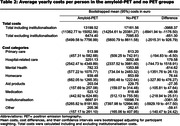# Long‐Term Impact of Amyloid PET in Memory Clinic Diagnostic Workup: A Matched Cohort Study on Healthcare Costs, Institutionalisation, and Mortality Risk

**DOI:** 10.1002/alz.086719

**Published:** 2025-01-09

**Authors:** Pieter J. van der Veere, Hana M. Broulíková, Jeroen Hoogland, Argonde C. van Harten, Ingrid S. van Maurik, Elsmarieke van de Giessen, Johannes Berkhof, Wiesje M. van der Flier

**Affiliations:** ^1^ Amsterdam Neuroscience, Neurodegeneration, Amsterdam Netherlands; ^2^ Alzheimer Center Amsterdam, Neurology, Vrije Universiteit Amsterdam, Amsterdam UMC location VUmc, Amsterdam Netherlands; ^3^ Department of Epidemiology and Data Science, Amsterdam UMC, Amsterdam Netherlands; ^4^ Vrije Universiteit Amsterdam, Amsterdam Netherlands; ^5^ Alzheimer Center Amsterdam, Amsterdam UMC, Amsterdam Netherlands; ^6^ Department of Radiology & Nuclear Medicine, Amsterdam UMC, Amsterdam Netherlands

## Abstract

**Background:**

We hypothesise that improved diagnostic precision, operationalised by adding amyloid positron emission tomography (PET) to the diagnostic work‐up in a memory clinic, is beneficial for long‐term health and healthcare cost outcomes. We investigated whether a more precise diagnosis influenced institutionalisation and mortality incidence trajectories, and annual healthcare costs over a period up to eight years.

**Method:**

Between October 2014 and December 2016, patients from the Amsterdam Dementia Cohort were offered an amyloid‐PET as part of their diagnostic work‐up. Those who received an amyloid‐PET were propensity‐score matched to participants without a PET, creating two balanced groups containing 442 patients each (Table 1; 64±8yrs, 40%F, 43% dementia). Statistics Netherlands provided healthcare costs for six years, institutionalisation for seven years, and mortality data for eight years of follow‐up. Institutionalisation and mortality incidence were calculated, and mean costs were bootstrapped. Healthcare costs were first evaluated as annual total cost and subsequently disaggregated into the following categories: primary care, hospital‐related care, mental health care, aid products, medication, homecare, and institutionalisation.

**Result:**

The amyloid‐PET and no‐PET groups had distinct institutionalisation and mortality incidence trajectories (Figure 1). The difference in institutionalisation was most pronounced three years after the diagnosis with 11% institutionalisation in the amyloid‐PET group (95% confidence interval [CI]: 8%‐14%, n=50), compared to 20% in the no‐PET group (15%‐25%, n=95, p=0.002). Subsequently, the difference attenuated to 28% (24%‐33%, n=118) vs. 33% (27%‐38%, n=135, p=0.28) after six years. Mortality incidences started to converge after four and merged after six years. Annual healthcare costs were €3968 (CI:1177‐6942, Table 2) lower in the amyloid‐PET group. This difference was mostly attributable to the categories of institutionalisation costs (€‐3361; CI:‐5929 to ‐918) and primary care (€‐99; CI:‐195 to ‐7).

**Conclusion:**

A more precise diagnosis through an amyloid‐PET positively influenced the incidence trajectories of institutionalisation and mortality, with the largest differences observed in the first three to four year after the diagnosis. Our findings suggest a more precise diagnosis could lead to more efficient and tailored primary care and to postponed institutionalisation. Less time spent in institutional care was also identified as a main driver of lower healthcare costs.